# Supplementary material for: An epitaxial graphene platform for zero-energy edge state nanoelectronics
Source: Nat Commun. 2022 Dec 19;13:7814. doi: 10.1038/s41467-022-34369-4 (PMC9763431; doi:10.1038/s41467-022-34369-4)
Supplement: Supplementary file 1 — Supplementary Information [file 41467_2022_34369_MOESM1_ESM.pdf]

Supplementary Information

SI1. *Charge puddles and residual conductivity in exfoliated graphene flakes*

A non-zero conductivity at CNP is observed in many graphene samples which normally manifests as a rounding at CNP: a linear fit beyond the rounding extrapolates to the baseline (σ=0) as in Fig. S1a. This rounding has been explained in terms of various forms of disorder (eg. charge puddles[1,2]). However, occasionally, a residual conductivity σ_{res} is also observed, where the extrapolation is significantly above the baseline (Fig. S1c, e, g). Since a residual conductivity is observed in exfoliated virgin graphene flakes (which have intact edges) but not in lithographically patterned exfoliated flakes the residual conductance is apparently caused by the edges, as it is in epigraphene ribbons, as explicitly demonstrated in the main text.

In their highly cited paper,[3] Chen, Fuhrer et al. experimentally demonstrated that charge impurity scattering causes the minimum conductivity and provided an empirical equation $\sigma(n)=Ce|n/n_{imp}|+\sigma_{res}$, where n is the charge density, n_{imp} is the impurity concentration and $C=5\text{x}10^{15}\ V^{-1}s^{-1}$. They cite Trushin et al.[4] who predict that charge impurity scattering will give a conductivity $\sigma(n)\approx\sigma_{res}(1+2\pi R^2 n)$, where R is the impurity screening radius and σ_{res} is proportional $1/(n_{imp} R^2)$. This explains σ_{res} but not the rounding.

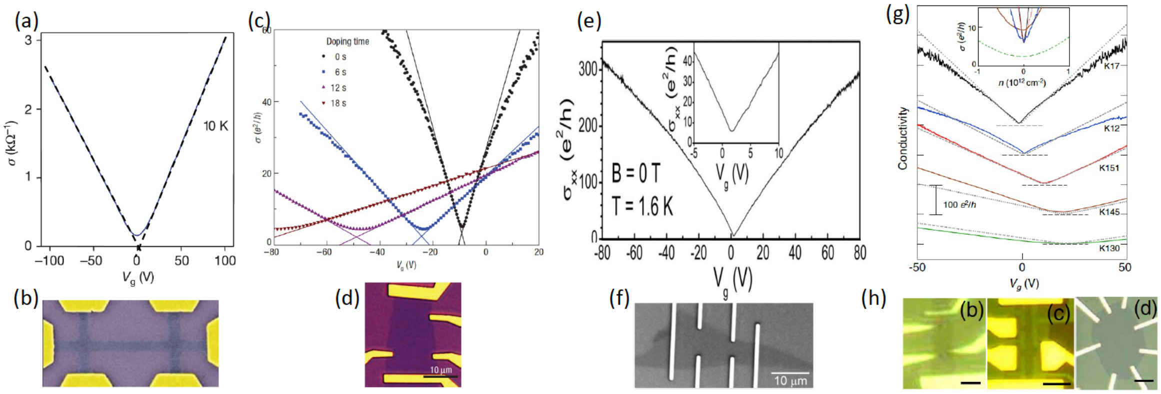

Figure S1. Conductivity as a function of gate voltage and corresponding optical images of the measured devices adapted from **(a)(b)** Ref. [5]; **(c)(d)** Ref. [3]: potassium doping of graphene for a pristine sample and three different doping concentrations taken at 20 K in UHV; **(e)(f)** Ref. [6]; **(g)(h)** Ref. [7]: the scale bars in the optical images in (h) represent 1 µm and 10 µm for (b) and (c),(d), respectively.

Cho and Fuhrer[6] concurrently provided compelling evidence that the rounding is due to relatively large P and N charge puddles and stated that σ_{res} is not understood (micron scale charge puddles are commonly observed in exfoliated graphene[2] but not in epitaxial graphene, see Fig.S2). They give several possible explanations for σ_{res} including experimental artifacts due to the non-ideal sample structures. In fact, in both papers[3,6] and for some of Kim et al's samples[7], the measured devices are

unpatterned exfoliated graphene flakes that are supplied with contacts (see Fig. S1d, f, h). In contrast the patterned Hall bar[5] from Novoselov et al. (Fig. S1a-b) does not have a residual conductivity.

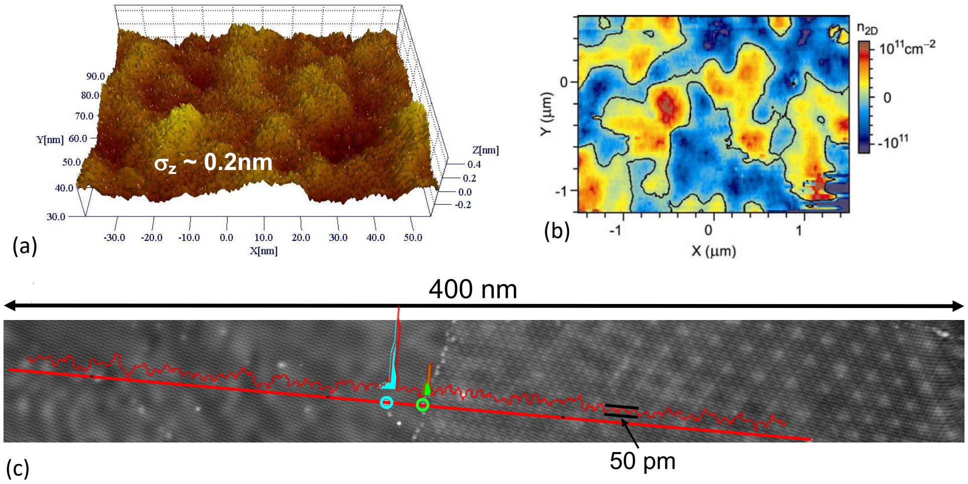

Figure S2. Surface characterization. **(a)-(b)** exfoliated graphene flake on SiO_2 substrate. **(a)** STM topographic image[8] **(b)** Color map of the spatial density variations in the graphene flake extracted from surface potential measurements at high density and when the average carrier density is zero. Blue regions correspond to holes and red regions to electrons.[2] **(c)** STM image of epigraphene (multilayer graphene on C-face), across a boundary between two different Moiré regions.[9] The layer is atomically flat with a roughness less than 50pm (red line - courtesy Joseph Stroscio).

The conductivity σ of a diffusive conductor is derived from the measured conductance G: if the conductance of a ribbon of length L and width W is G, then its conductivity is σ=GL/W, which is independent of L and W. It has been shown that inhomogeneous graphene (i.e. with charge puddles) and with charged impurities has a non-zero conductivity at the charge neutrality point: σ(CNP)≠0. Nevertheless the conductance scales with L: σ(CNP)=G(CNP)L/W for measurements at several lengths L on the same sample. Therefore, ballistic transport at CNP cannot be concluded simply from a non-zero conductance at CNP (even if it appears to be quantized). An unambiguous way to demonstrate a ballistic edge state is to measure conductance G(CNP) at several lengths L to show that $G(CNP)=G_e(1+L/\lambda)^{-1}$. In this way, we have definitively demonstrated that in our samples the residual quantized conductance G_e is due to the edge state and that it cannot be incorporated in the conductivity of the sample because of length scaling.

It may well be that the edge state in fact did contribute to the conductance in many of the early, unpatterned graphene flakes but this possibility was not investigated and a residual *conductivity* was assumed, which had different values on samples produced from the same unpatterned flake but with different geometries[7] which is in fact a sign of a ballistic edge state. On the other hand, lithographically patterned exfoliated graphene flakes like Fig. S1a have disordered edges and therefore have no edge state and no residual conductance so that this problem went away when patterned samples were used.

SI2. ***Corbino ring measurements***

> **A Corbino ring is an edgeless device specifically designed to eliminate properties caused by sample edges. Therefore differences in properties observed in a Corbino ring and in a Hall bar can be attributed to the edge state. These measurements show that the residual conductance, the anomalous magnetic properties, and the anomalous temperature dependence observed in the epigraphene ribbons are all due to the edge state, consistent with the conclusions in the main text.**

The Corbino ring was produced using the process below (Fig. S4). It consists of a conducting ring with electrodes contacting its inner and outer rims (Fig S3a-d). Comparing the transport in the Corbino ring and that of the Hall bar, we can further separate the transport properties related to the edge from those of the bulk.

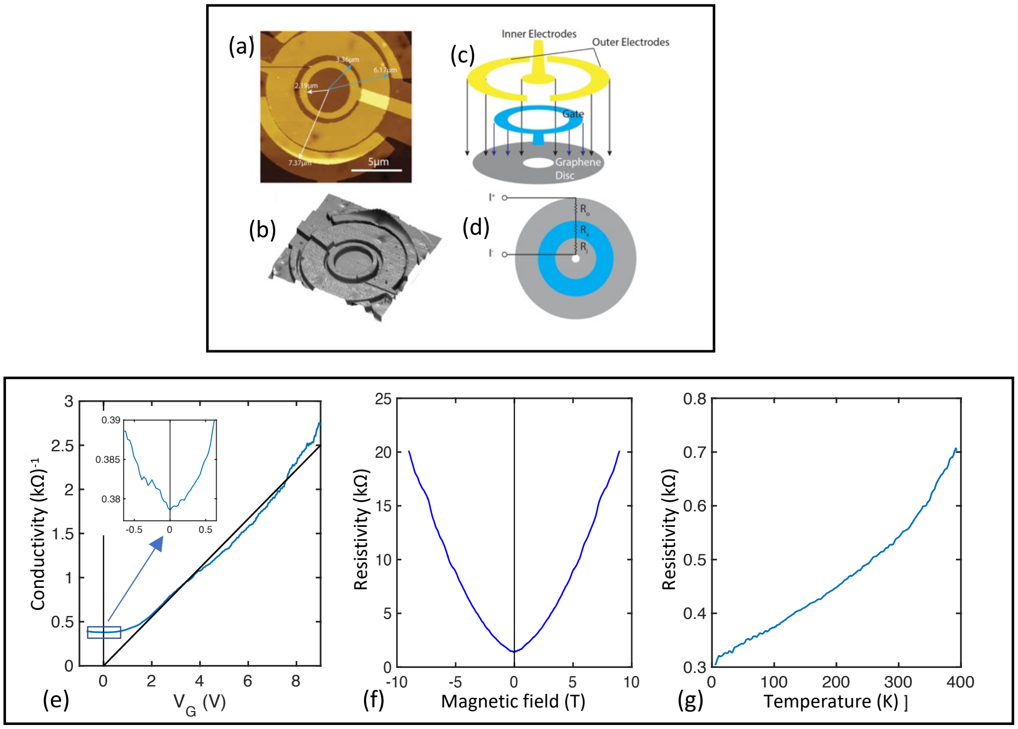

Figure S3. Corbino ring. **(a)** AFM image of the Corbino ring. **(b)** 3d rendering of the device. **(c)** Construction diagram showing electrodes (yellow), Al_2O_3 dielectric (blue), graphene (gray). **(d)** The Corbino ring is modeled as three resistors in series. **(e)** Conductivity at 4.5K as a function of gate voltage showing a significant non-zero minimum conductivity at CNP, however there is no significant residual conductivity, in contrast to that observed in the S1 and S2 Hall bars where it is due to the edge state. **(f)** A large positive magnetoresistance is observed at 4.5K. In contrast, the S1 and S2 Hall bar resistance decreases with increasing magnetic field. **(g)** The bulk resistivity increases with increasing temperature. In contrast the S1 and S2 Hall bar resistance decreases with increasing temperature (see SI9).

Figure S3e shows the conductivity σ versus gate voltage. A minimum conductivity, which properly extrapolates to $\sigma=0$ at CNP, is observed. In contrast to the Hall bar, the Corbino ring does not have a significant residual conductance.

Figure S3f shows the large *positive* magnetoconductance, which is consistent with $\rho=(1+(\mu B)^2)/ne\mu$ with $\mu=4300$ $cm^2V^{-1}s^{-1}$ and $n=1.2x10^{12}$ cm^{-2} at $V_G=2.5$ V. In contrast, the Hall bars S1 and S2 have a large negative magnetoconductance that saturates at about B=3T, which therefore can be attributed to the edge state.

The graphene to Pd/Au contact resistance is 890 Ω, similar to the S1 and S2 Hall bar contact resistance. Similarly to the S1 and S2 Hall bars, charging due to the Al_2O_3 dielectric is observed: $n_0 \approx 3x10^{12}cm^{-2}$.

Figure S3g shows the resistivity of the Corbino ring at $n=4x10^{12}$ cm^{-2} as function of temperature, showing an approximately linear increase for T<200 K, followed by a non-linear increase at higher temperatures, consistent with scattering from acoustic phonons.[10] In contrast the resistance of the Hall bars *decreases* with increasing temperature so that this property also can be attributed to the edge state (see SI9).

Hence, these measurements show that the edge state causes the residual conductance, the anomalous magnetic properties, and the anomalous temperature dependence.

SI3. *Device production*

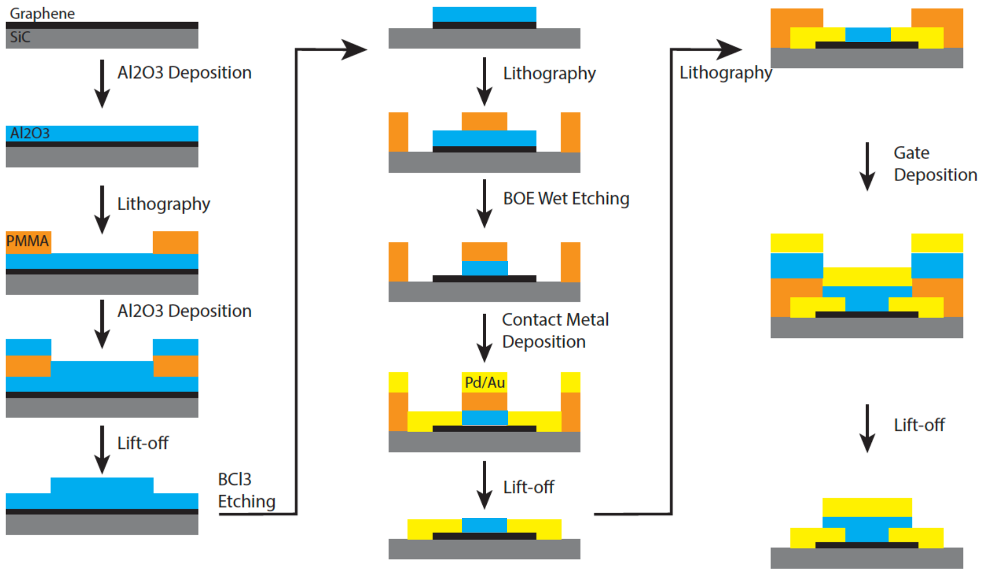

Figure S4. Device production process flow Sample S1 (Method 1).

Hall bar S1 was produced using the process flow described in Fig. S4, however the dielectric patterning failed in the first attempt and was removed using hydrogen fluoride (HF), after which it was reapplied. In this process the graphene was subjected to hydrogen fluoride and this Buffered Oxide Etch wet etching significantly reduced the graphene mobility. This turned out to be fortuitous because it greatly enhanced the contrast between the edge state transport and the bulk transport. Moreover, it further demonstrates that the edge state is not affected by disorder that greatly reduces the mobility of the bulk. This immunity of the edge state to processing damage is important for nanoelectronics fabrication.

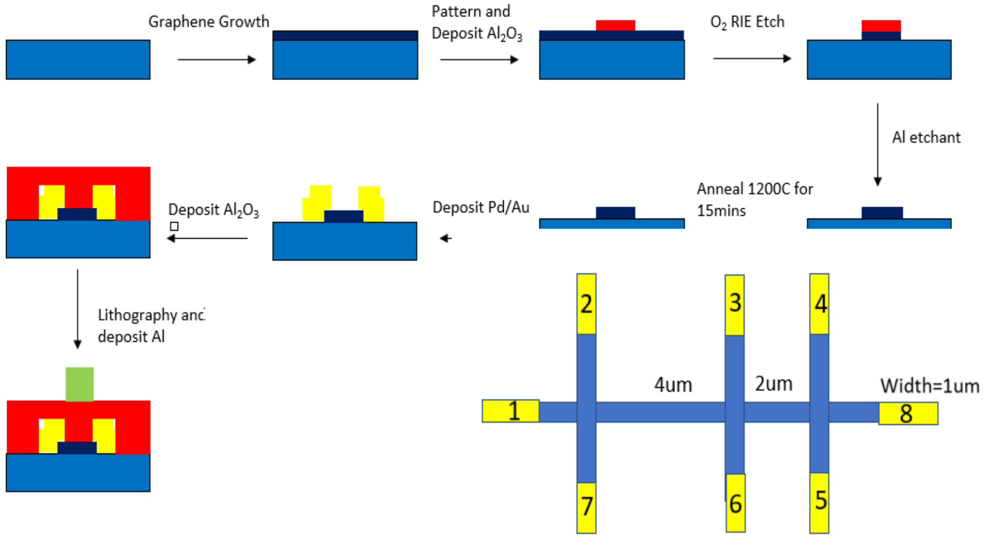

Figure S5. Device production process flow for Sample S2 (Method2)

Hall bar S2 was produced using the process flow described in Fig. S5. The process is simplified compared with Sample S1 (Fig. S4). A patterned Al_2O_3 layer was used as a mask for the oxygen plasma etch. The mask was removed with Al etchant and the patterned graphene was annealed at 1200°C for 15 min. Pd/Au contacts were then deposited and the Al_2O_3 dielectric was deposited followed by the deposition of the Al gate electrode.

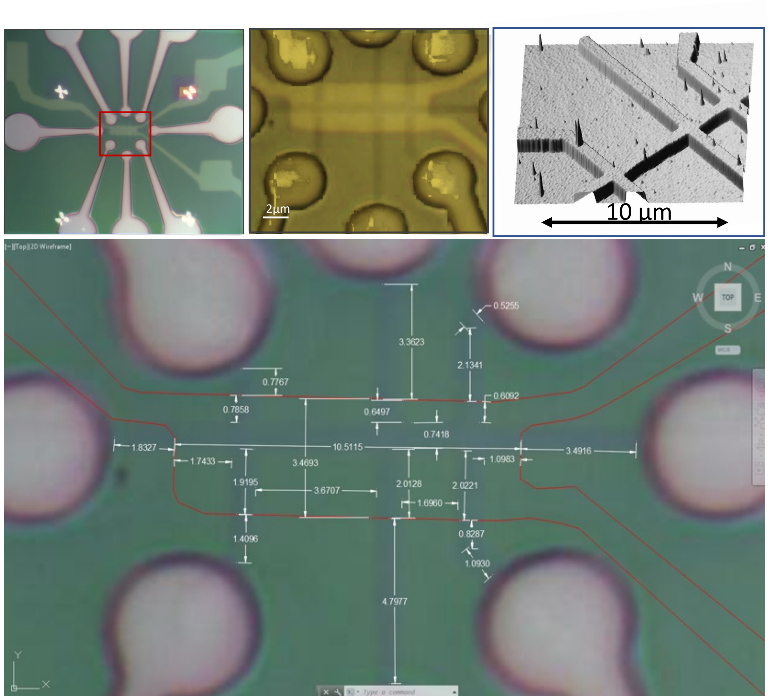

Figure S6. *Optical images of the Hall bar S1.* (Top left) Superposition of an image taken before gate deposition, with the resist in place, to outline the geometry of the Hall bar, and an image of the completed device. (Top middle) zoom in of the red square in the left image. (Bottom) Dimension measurements. Red line indicates the perimeter of the gate. (Top right) 3D rendition of the AFM image of sample S1.

SI4. *Partially gated graphene ribbons*

> **Partially gated graphene ribbons show that the edge state conductance is not affected by the bulk charge density step at the interface between gated and ungated portions of the ribbon segment. They also show that the bulk conductivity along the ZZ and AC directions are identical, however that the edge state mfp along the ZZ direction is much larger than along the AC direction.**

Graphene ribbons that are partially gated have been measured using 2-point and 3-point measurements (4-point measurements are for fully gated segments). For these, the conductance of the bulk component consists of two conductors in series: G_{gated}^{bulk} and $G_{ungated}^{bulk}$ corresponding to the measured gated and ungated segment lengths L_{gated} and $L_{ungated}$. Hence the net conductance of a single segment after subtraction of the residual conduction (i.e. the edge state) is

$$G_x^{bulk} = \left(\frac{L_{gated}}{W}\frac{1}{\sigma(n)} + \frac{L_{ungated}}{W}\frac{1}{\sigma(n_0)} + R_{contact}^{bulk}\right)^{-1} \qquad \text{Eq.S1}$$

Where σ(n) is determined from the 4-point measurements and $\sigma(n_0)$ is the ungated conductivity. The edge state conductance is G_x^{edge}, see Eq. 2 in main text:

$$G_x^{edge} = 1/\left(R_0(1 + L/\lambda) + R_{junc} + R_c\right) \qquad \text{Eq.S2}$$

So that the total conductance is (Eq. 1)

$$G = G_x^{edge} + G_x^{bulk} \qquad \text{Eq.S3}$$

As shown in Fig. S7, Eq. S1 applied to 2, 3, and 4 point measurements gives excellent agreement for $\sigma(V_G)$ over the entire V_G range, using $\sigma(n_0)$=2.7 G_0 (n_0=1.2x10^{12}cm^{-2}) and $R_{contact}^{bulk}$=0.015 R_0 (≈400 Ω). However if the residual conductance is not subtracted there is no agreement at all. This again clearly demonstrates that the residual conductance cannot be lumped together with the minimal conductivity, since this combination will not property scale with length. It also shows that the residual conductance does not depend on V_G.

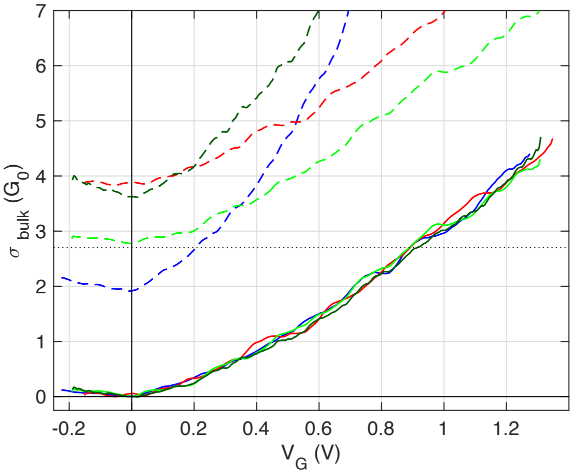

Figure S7. Solid lines show bulk conductivity (Eq. S1) after subtraction of the residual conductance, taking into account the ungated lead resistance and contact resistances (4.5K measurements). The conductivities superimpose showing excellent agreement for 2, 3 and 4 point measurements. Seg. **A**: $G_{15,12}$, 3-point (blue); Seg. **B**: $G_{15,23}$, 4-point (red); Seg. **C**: $G_{15,34}$, 4-point (green); Seg. **A**+**B**+**C**+**D**: $G_{15,15}$, 2-point (black). If the residual conductance is not subtracted (dashed lines) then no scaling factor can superimpose them which unambiguously demonstrates that the residual conductance does not scale like a conductivity and that it does not depend on the gate voltage.

These measurements confirm that the bulk conductivity is the same in different ribbon regions, as expected for a homogeneous material; it is explicitly identical along the ZZ and the AC directions: $\sigma_{AC}=\sigma_{ZZ}$. It also shows that the edge state conductance is not affected by the bulk charge density step at the interface between gated and ungated portions of the ribbon segment.

SI5. *Magnetoconductance dip*

A broad dip (width≈3 T) in the magnetoconductance at B=0 is observed in epigraphene ribbons and in sidewall ribbons, however not in Corbino ring measurements. The dip vanishes at increased temperatures. The dip is associated with edge state scattering at junctions, contacts and edge defects.

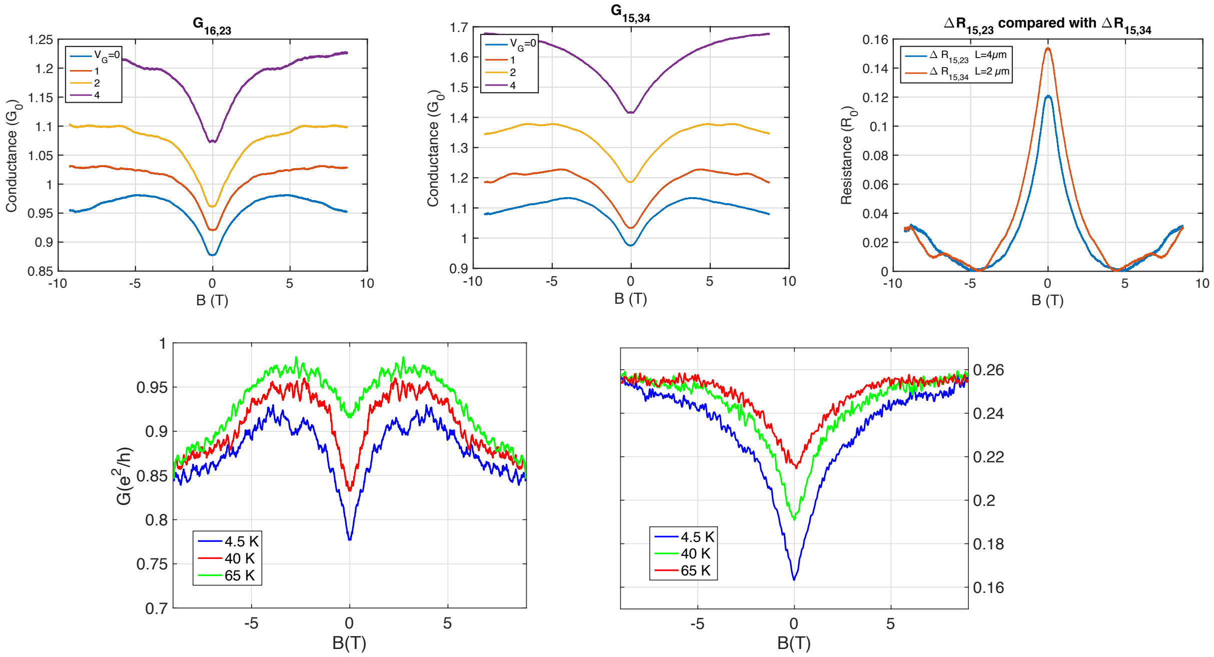

Figure S8. The conductance of graphene ribbons with an edge state shows a dip in the magneto-conductance at B=0 and 4.5K (top panels and for various temperature (bottom panels). **Top left panel** is $G_{15,23}$ (Sample S2-Seg. **B**, 4µm, ZZ segment, 4-point), **top middle panel** is $G_{15,34}$ (Sample S2-Seg. **C,** 2 µm long ZZ segment, 4-point) measured for 3 gate voltages. **Top right panel** compares the resistance of the dip $\Delta R=R(B)-R_{min}$ at $V_G=0$ of $R_{15,23}$ and $R_{15,34}$; $\Delta R_{15,23}$ and $\Delta R_{15,34}$ are almost identical, indicating that the dip is primarily caused by scattering of the edge state (as it is observed at CNP) at the junction and not due to scattering along the ribbon because the resistance difference ΔR would scale linearly with the length difference ΔL=2 µm. If the small residual ΔR=0.033 R_0 is attributed to ΔL, then ΔR corresponds to a mean free path $\lambda_{ZZ}=R_0\Delta L/\Delta R=60$ µm, which is consistent with other measurements (see main text). **Bottom left panel** is $G_{15,45}$ (Sample-S1, Seg **D**, 4.5 µm, AC direction, symmetrized, 3-point) at CNP; it shows the decrease of the dip with increasing temperature and the saturation near $G=G_0$. All individual segments show similar behavior. **Bottom right panel**, same measurement but for $G_{15,15}$ (Sample S1-Seg. **A+B+C+D**, 15 µm, AC direction, 2-point). The conductance saturates at $G_0/4$, consistent with four 1 G_0 ribbon segments in series. Note that the dip amplitude is considerably larger along the AC direction than the ZZ direction, indicating that scattering at AC edge defects and at the junctions is reduced both in a magnetic field and at increased temperatures.

SI6. *Pseudo-plateaus*

Non-quantized quantum Hall resistance plateaus are observed close to CNP that saturate at about R≈0.25 R_0 for $|V_G|$>0.3 V. The anomalous ≈0.25 R_0 quantum Hall plateau and the pseudo plateaus are caused by the shunting effect of the edge state.

Near CNP, the Hall resistance of sample S1 measured for various gate voltages V_G, has non-quantized plateaus that converge to ≈0.25 R_0 for V_G=0.3 V as shown here for $G_{15,26}$, Sample S1. The pseudo-plateaus converge to R_H=0 at CNP, rather than diverging which is caused by the shunting effect of the edge state. The value of the plateau is similarly explained by the shunting effect.

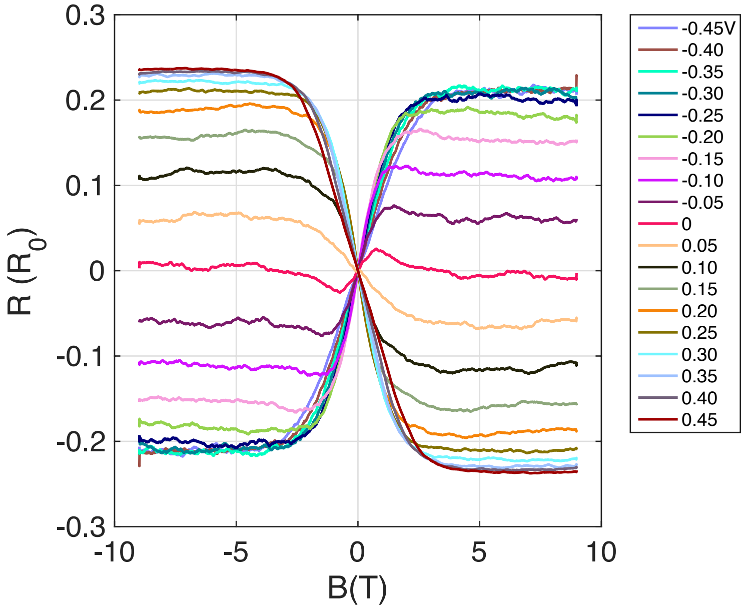

Figure S9. Resistance $R_{15,26}$ (Hall measurement configuration at 4.5 K) of sample S1 for various gate voltages, showing non quantized plateaus for B>3T.

SI7. ***Schematic diagram of the 1D edge state network***

The edge state forms a 1D network that can be schematically represented by ballistic segments with a resistance of 1 R_0 between contacts and junctions.

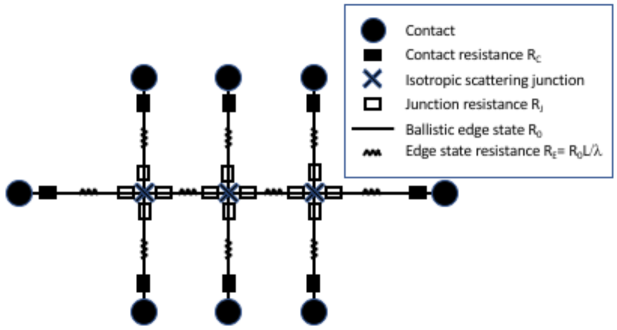

Figure S10. Schematic diagram of the 1D edge state network of S1 and S2.

The resistance is increased by the junction resistance R_J, the contact resistance R_C, and scattering along the edge state, $R_E=R_0L/\lambda$ so that each segment has an effective resistance $R_{Seg}=R_0+R_E+R_J+R_C$. Note that R_E is larger for the AC direction compared with the ZZ direction, whereas defects at segment corners probably cause R_J. Both R_E and R_J reduce in a magnetic field and at increased temperatures (see above). The effects are reminiscent of weak localization. The large width of the magnetoconductance dip may be due to the 1d nature of the edge state that cannot enclose a magnetic flux.

SI8. ***Two component Hall effect***

The edge state network and the bulk network are independent and only interact at the contacts. Therefore the measured Hall voltage is a combination of the edge state Hall voltage and the bulk Hall voltage that involve currents in the voltage arms of the Hall bar.

We have shown that the edge state network and the bulk network are independent and only interact at the contacts. This also applies to the Hall voltage, as shown in Fig. S11, where the bulk network is labeled 1 and the edge state network is labeled 2. A current I_0 is applied between contact A and B and is divided between the two networks: $I_{1,2}=I_0G_{1,2}/(G_1+G_2)$ where $G_{1,2}$ are the conductances. These currents generate Hall voltages $V_{1,2}=R_{H1,2}I_{1,2}$ that cause a current i to circulate along the vertical arms of the Hall bar: $i=(V_1-V_2)/(R_1+R_2)$, so that the resulting measured Hall voltage $V_{CD}=V_1-iR_1=I_0R_{H1}(R_1/R_2+1)^{-1}(G_2/G_1+1)^{-1} + I_0R_{H2}(R_2/R_1+1)^{-1}(G_1/G_2+1)^{-1}$. For a diffusive conductor $R_{H1}=B/en$. For a chiral 1 G_0 edge state $R_{H2}=R_0$. For the edge state we find that $R_{H2}=0$. For a cross of equal arm lengths $G_1=1/R_1$ and $G_2=1/R_2$, which results in Eq. 3a in the main text; setting $R_{He}=0$ gives Eq. 3b, main text.

$R^{m}_{Hall} = R_{Hb} + R_{He} = \frac{B}{ne}(R_b/R_e + 1)^{-2} + R_0(R_e/R_b + 1)^{-2}$ (Eq. 3a, main text)

$R^{m}_{Hall} = \frac{B}{ne}(R_b/R_e + 1)^{-2}$ $(R_{He} = 0)$ (Eq.3b, main text)

On the other hand, for a cross with equal arms in the quantum Hall limit of the LL_0 state, we expect for the edge state, $R_1=R_e=R_0$; $G_1=G_e=G_0$ and $R_2=1/G_2=R^{0Dis}$ where G_2 is the measured 2 point conductance (note that in the quantum Hall regime the Hall resistance equals the two point resistance). With these substitutions and assuming that the Hall voltage of the flatband vanishes, we find Eq. 4 of the main text:

$R^{Pred}_{Hall} = R^{0Dis}\left(R^{0Dis}/R_0 + 1\right)^{-2}$ (Eq.4, main text)

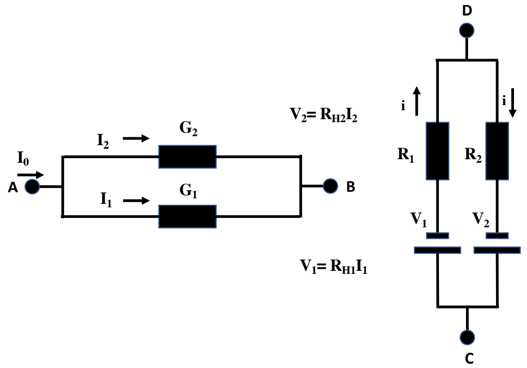

Figure S11. Schematic diagram voltages and currents in the interacting edge state and bulk networks for a 4-contact cross A-B-C-D where a current I_0 is applied horizontally along AB, generating Hall voltages $V_{1,2}$ in the vertical arms (CD).

SI9. ***Temperature dependence***

The conductance of epigraphene ribbons *increases* with increasing temperature, rather than *decreasing* as in Corbino ring measurements and for 2D graphene in general, which shows that the conductance increase is an edge state property. In a magnetic field this increase is suppressed (see below).

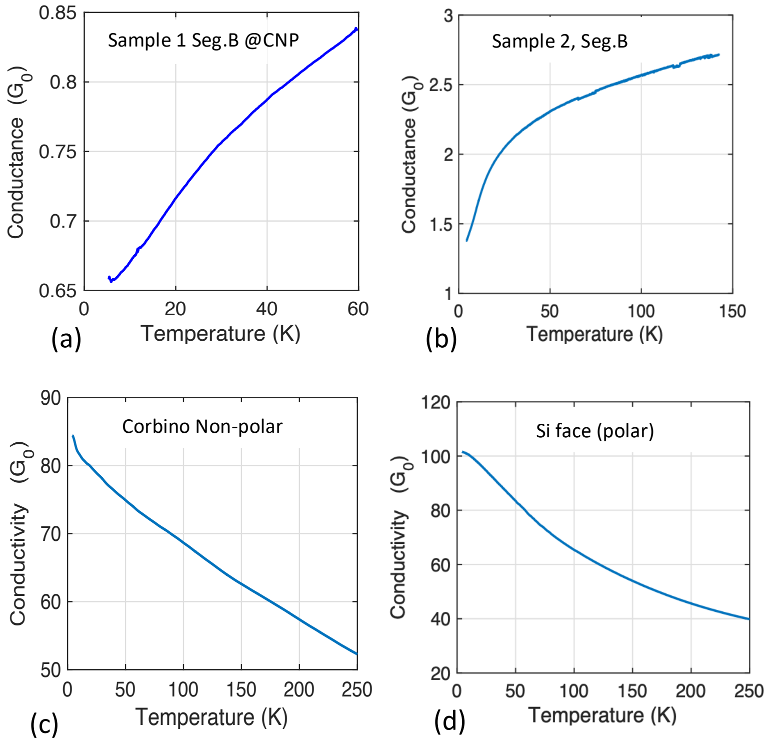

Figure S12. Temperature dependences. **(a)** Conductance of Sample 1-Seg. B (L=4 µm) measured at V_G=0; **(b)** Conductance of Sample 2-Seg. B (L=4 µm), measured at V_G=4V; **(c)** Corbino ring conductivity of non-polar epigraphene; **(d)** conductivity of Si face, polar 2D epigraphene.

SI10. *High temperatures and the excited edge state*

High temperature measurements show that the anomalous quantum Hall plateau persists and is consistent with the edge state shunted $R_0/2$ quantum Hall plateau that is derived from the high mobility N_{0Dis} subband. The anomalous temperature and magnetic field dependence is consistent with a 1 G_0 excited edge state on the order of 10 meV above the ground state, with ballistic properties that resemble the ground state. This state can only be accessed thermally and not with a gate voltage, which implies that it is located at the edge that is pinned at E=0, making it insensitive to V_G. The excited state is suppressed in a magnetic field.

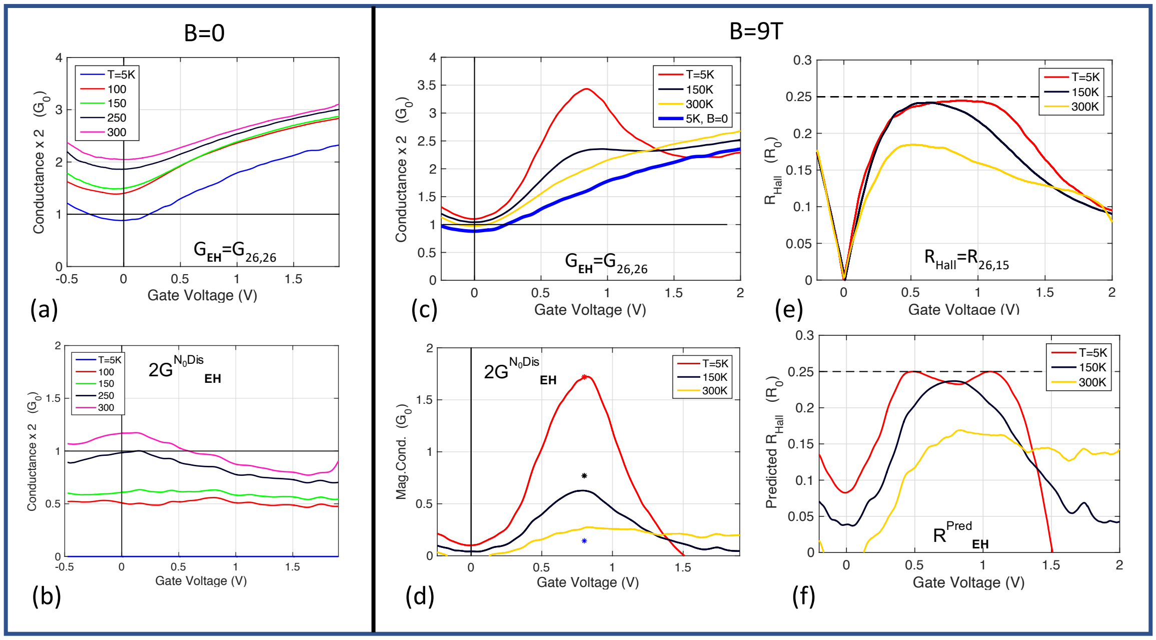

Figure S13. *Excited edge state and N_{0Dis} for the 2 segments **E+H** inseries, Sample 1, 2points* **(a)** $2G_{EH}$ at B=0 T for T=5 K, 50 K and 300 K (the factor 2 accounts for 2 segments in series like in Fig. 4e, main text). **(b)** $2G^{N0Dis}(T,V_G)$ at B=0 T for T=5 K, 50 K and 300 K, where $G^{N0Dis}(T,V_G)=G_{EH}(T,V_G)-G_{EH}(T=5K,V_G)$ Note that G^{N0Dis} increases essentially uniformly with increasing temperature suggesting an excited edge state of the edge state with a conductance of essentially 1 G_0. **(c)** $2G_{EH}$ at B=9 T for T=5 K, 50 K and 300 K. **(d)** $2G^{N0Dis}(T,V_G)$ at B=9 T for T=5 K, 150 K and 300 K. The edge state conductance reverts to ≈1 G_0 indicating that the excited state is suppressed in a magnetic field. The Shubnikov -de Haas oscillation has a maximum conductance of about 2 G_0, i.e. consistent with the conductance of LL_0. The reduction with increasing T is consistent with the Shubnikov-de-Haas peak of the LL_0 state of graphene (E_{LL0}/k_B =1250 K at B=9 T) as indicated by the asterisks. **(e)** Hall resistance $R_{Hall}=R_{26,15}$ at T=5 K, 150 K, 300 K showing the anomalous plateau persists at high temperatures, consistent with a high mobility N=0 dispersive graphene subband. **(f)** Hall resistance as predicted in Eq.4 of the main text, using measured G^{N0Dis} plotted in (d) as in Fig.4g of the main text.

Figure S13a shows $2G_{EH}=2G_{26,26}$ for several temperatures from 5 K to 300 K at B=0 T. Figure S13b shows $2G_{EH}(V_G,T)-2G_{EH}(V_G,T=5K)$ which corresponds to the conductance increase with increasing

temperature. Note that $G_{EH}=(G_E^{-1}+G_H^{-1})^{-1} \approx G_{Seg}/2$ i.e. half of a single segment conductance which is the reason for the factor of 2 in the y axis.

Since the bulk conductance only weakly decreases with increasing temperature, this increase is due to the edge state (see Corbino data, SI2). Note that the increase is close to 1 G_0 and relatively independent of V_G, which is similar to the low temperature edge state behavior. Since this behavior is not seen in sidewall ribbons, we can reasonably assume that an excited state is involved with an energy gap on the order of 10 meV. This gap value is consistent with the observed 0.1 eV gap observed in 40 nm wide sidewall ribbons[11] assuming a 1/W width dependence, which is often observed in calculated energy gaps in edge states. Moreover, the fact that the edge state conductance at high temperatures is 2 G_0 is consistent with a 2-fold degenerate edge state as predicted for the edge state in general.

Figure S13c shows $2G_{EH}(V_G,T, B=9T)$ Note that now the conductance at CNP only slightly increases with increasing temperature which is explained in the main text as due to reduced scattering at the junction. If we subtract the conductance at T=5K, B=0: $G_{EH}(V_G,T,B=9T)-G_{EH}(V_G,T=5K,B=0T)$, then we recover the temperature and field dependence of the N_{0Dis} (as explained in the main text) which shows a clear Shubnikov de Haas oscillation that corresponds to LL_0 (see main text).

The amplitude $A_{SdH}(B,T)$ of the G(B,T) oscillations (Shubnikov-de Haas oscillations) are given by the Lifshitz-Kosevich equation: $A_{SdH}(B,T)$ is proportional to u/sinh(u), where $u=2\pi^2 k_B T/E_{LL1}(B)$ and $E_{LL1}(B)$ is the energy of the LL_1: $LL1(B) = v_F c^* \sqrt{2e\hbar B}$. Hence, normalizing to G_{EH}(T=5K, B=9T) gives A_{SdH}(T=150 K, B=9 T)=0.75 G_0 and A_{SdH}(T=300 K, B=9 T)=0.15 G_0 which corresponds well with the observed amplitudes (Fig. S13d), leaving little doubt that this is the correct interpretation.

Figure S13e shows the measured Hall resistance showing the anomalous Hall plateau associated with LL_0 (see main text) for B=9T at various temperatures. Note that the Hall plateau is still visible at T=150 K, and is decreased at T=300 K. This trend is expected for LL_0 however the anomalous value (i.e. 0.25 R_0 rather than 0.5 R_0) implies that the shunting due to the edge state persists up to high temperatures. This interpretation is confirmed in Fig. S13f which shows that the Hall resistance continues to be well predicted from the measured G^{N0Dis} (Fig. S13d) using Eq.4 (main text) up to T=300 K.

SI11. *Resistance segmentation and quantization*

Resistance (in units of $R_0=h/e^2$) measured at CNP closely equals the number of segments, so that the resistance per segment is very close to 1 R_0 (at most 12% above R_0 at B=0) for all the segments measured. For B>0, the resistance decreases, resulting in essentially exact 1 R_0 quantization at high field, reminiscent of decreased coherent backscattering in magnetic field.[12] Similarly, at CNP and B=9 T, $R_E \approx 1\ R_0$, $R_{E+H} \approx 2\ R_0$ for all temperatures studied. Hence, at CNP the resistance per segment is approximately 1 R_0 at all temperatures both for nearly zigzag and armchair segments.

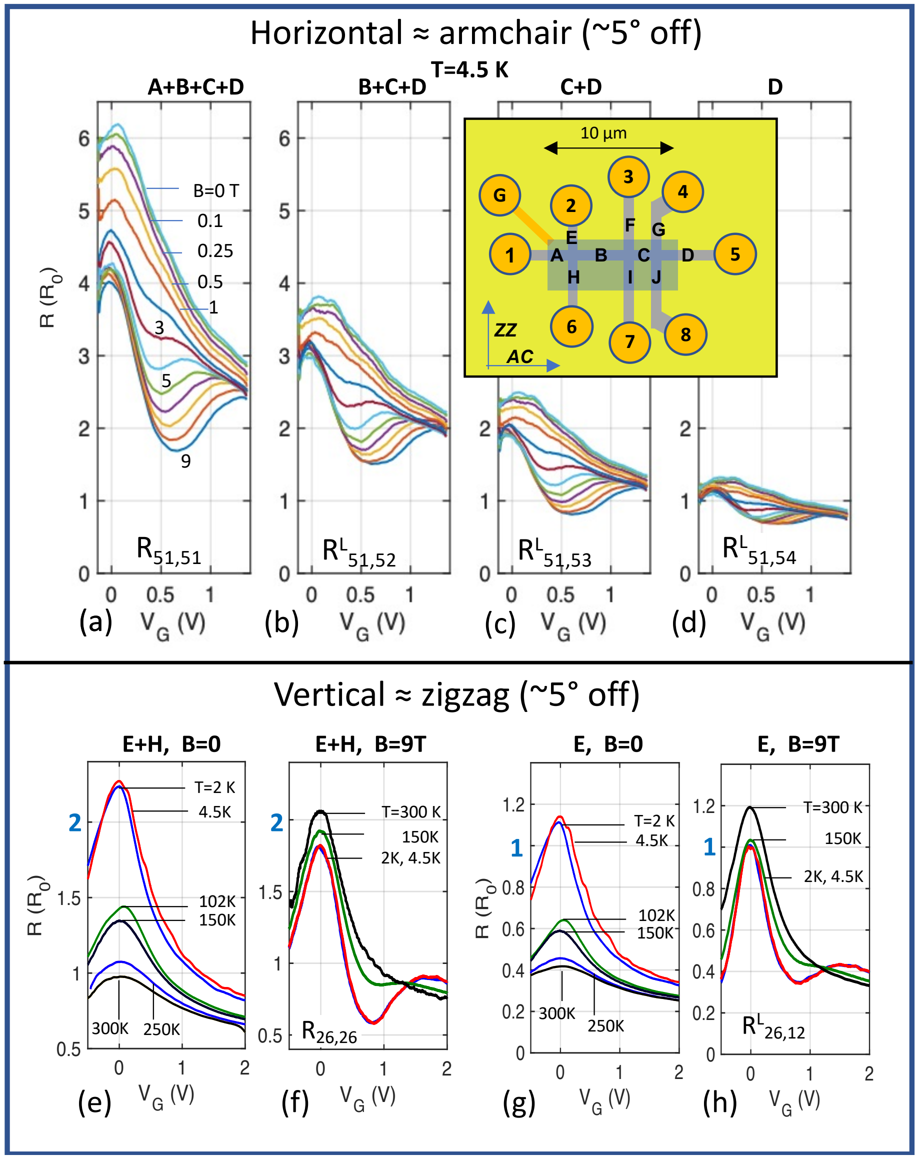

Figure S14. *Quantization and segmentation of the resistance.*

Three-point and two-point resistance (in units of R_0=h/e^2) for sample S1, width=740 nm, armchair orientation. $R^L_{ij,kl}$ denotes the longitudinal resistance R^L=(R(B)+R(-B))/2 with current flowing from contact i to j, and voltages measured between contacts k and l (**a**)-(**d**) *Horizontal segmentation*. $R^L_{51,5X}$ (X=1, 2, 3, 4) of the horizontal segments **A**, **B**, **C**, **D** (T=4.5 K, magnetic field |B| from 0 to 9 T and V_G from -0.1 to 1.3 V). At CNP and |B|≥2 T, $R^L_{51,5X}$ = 4, 3, 2, 1 R_0 for X=1, 2, 3, 4 demonstrating 1 G_0 conductance quantization of the conductance in the segments and scattering at the junctions. At CNP and |B|≤2 The resistance increase is caused by reduced backscattering, reminiscently of coherent backscattering[12] **(e)-(h)** *Vertical segmentation*. **(e) (f)** segments **E+H** ($R_{11',11'}$); **(g) (h)** segment **E** ($R^L_{11',01}$). For **(e) (g)** B=0 T: resistance reduction at CNP with increasing temperature is due to thermal population of the bulk subbands. **(f) (h)** B=9 T: Edge state resistance quantization at CNP is observed up to T=300 K. Inset shows the device composed of a 15 μm long 740 nm wide horizontal ribbon crossed with 3 vertical ribbons giving 10 segments (white letters), 3 junctions, 8 low resistance Ohmic Pd-Au contacts. Measured segment lengths, in μm: L_A=3.6; L_B=3.3; L_C=1.7; L_D=4.5; L_E=1.6; L_F=4.0; L_G=3.3; L_H=3.6; L_I=6.8; L_J=3.8; (see Fig. S4). Vertical is 5° from the zigzag edge direction. The nominal gate efficiency (shaded rectangle) is dn_c/dV_G=-0.9x10^{12} V^{-1}cm^{-2}. Gate voltages V_G are reported with respect to CNP.

SI12. *Supplemental structural and spectroscopic characterization of monolayer non-polar epigraphene*

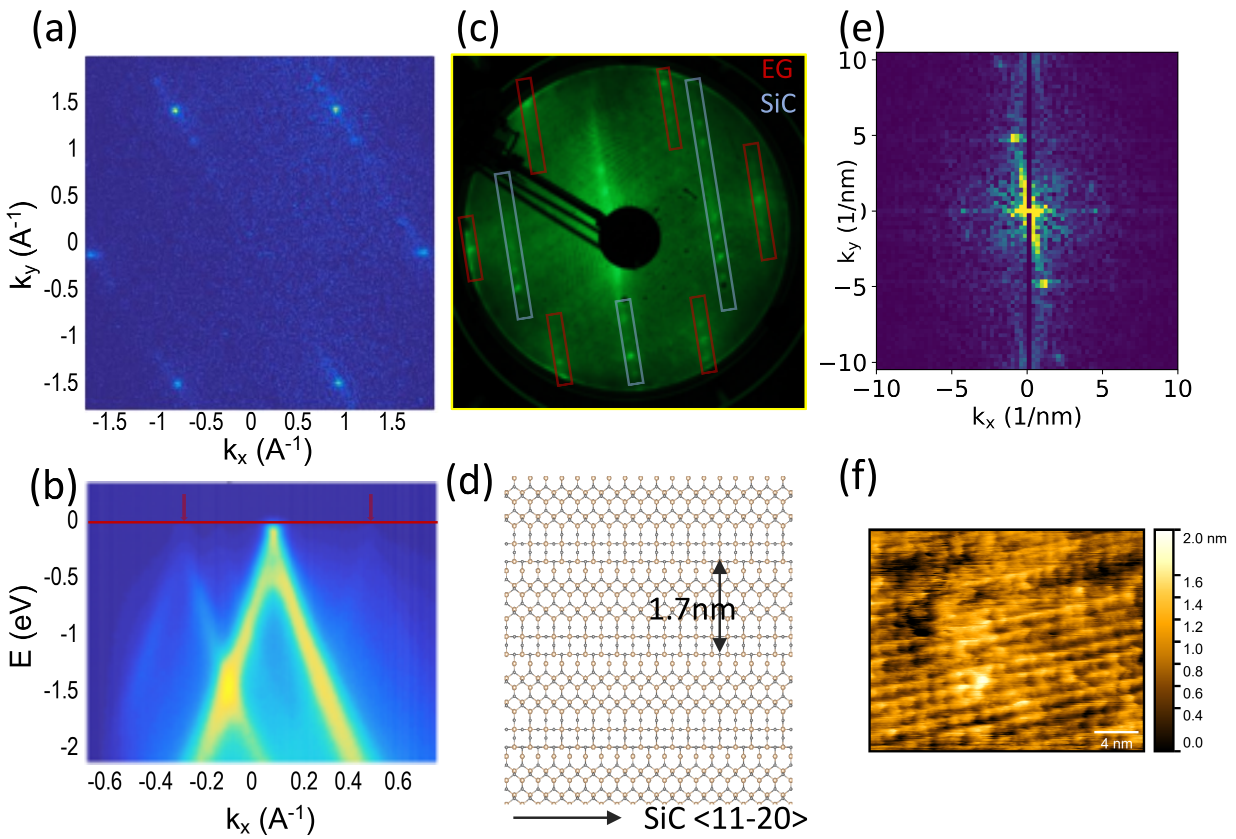

Figure S15. Sample S4 (non-patterned 2d epigraphene grown on non-polar facet 4HSiC$(1\bar{1}0n)$, n≈5) presents characteristics of monolayer graphene, with a Raman 2D peak at 2758 cm^{-1} (λ_{laser}=532 nm) that may indicate interaction with the substrate. **(a)** Fermi surface measured by ARPES (Cassiopée beam line at the Soleil synchrotron) at room temperature at E=0 eV (Beam energy 200 eV, E_F=197.4 eV), showing the expected hexagonal Brillouin zone for graphene. No distortion of the Brillouin zone is observed. The Dirac point is at E_F; replicas are observed in one direction only, with periodicity 0.4±0.01 Å^{-1}, consistent with LEED. Note that the k_x axis is oriented 30° from the <11-20> SiC direction shown in (d). **(b)** Energy vs k_x map at k_y=1.624Å^{-1} from ARPES measurements (beam energy=3 eV, E_F=32.1 eV); the sample was rotated so that the k_x axis is oriented along the replica dots, i.e. perpendicularly to the <11-20>SiC direction shown in (d). The plot shows the linear graphene dispersing band and the Dirac point at E_F=0 (red line) and two (faint) replica band on each side (red arrows). **(c)** LEED pattern (E=73 eV) showing the graphene (outlined by red rectangles) and SiC (outlined by light blue rectangles) diffraction spots. Rectangles are oriented perpendicularly to the <11-20> SiC direction shown in (d). Replica spots aligned with the graphene indicate uniaxial modulation of the graphene by the substrate. The spot separation Δk=0.39±0.02 Å^{-1} agrees with ARPES. **(d)** Example of the SiC (1-105) facet (top view), that presents a periodicity of 1.7 nm perpendicular to the <1-105> direction. **(e)** Fast Fourier transform of the STM image in (f) showing two main peaks corresponding to a modulation of periodicity 1.3±0.26 nm. **(f)** STM image (bias voltage =90 mV, I =900 pA). The parallel set of lines is along the <1-10n> direction, as expected from (d).

SI13. ***Spin polarization in sidewall nanoribbons***

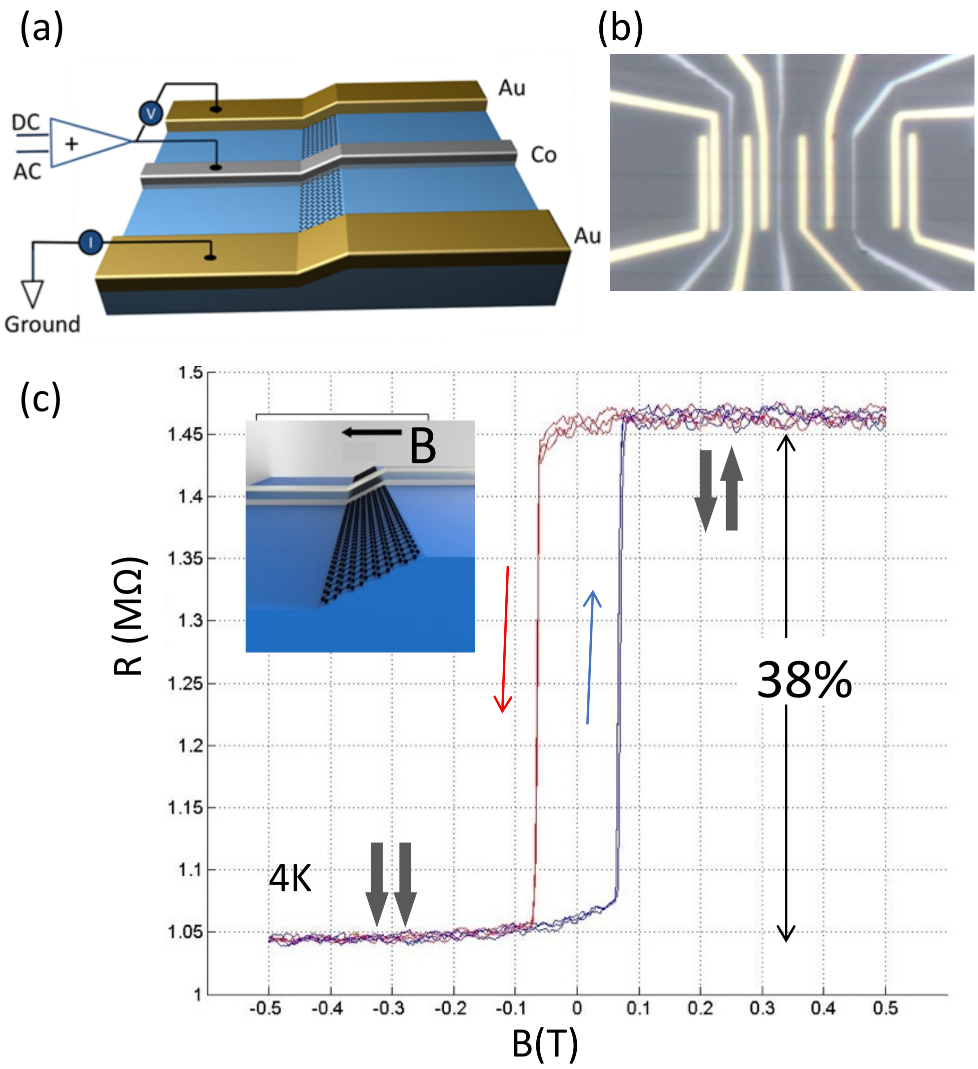

Figure S16. *Epigraphene sidewall nanoribbon spin polarized transport* (from Refs [13,14]). **(a)** Schematics of a three terminal non-local device, including a slopping epigraphene sidewall nanoribbon connected with a single spin polarizing tunnel contact (Cobalt on Alumina) and two Pd/Au contacts. An AC+DC voltage is applied between the tunnel contact and a Pd/Au contact, where current is measured, while the non-local voltage is measured on the opposing Pd/Au contact. **(b)** Optical image of a multi-terminal device (grey: Co/Al_2O_3, gold: Pd/Au). **(c)** Reproducible non-local tunnel resistance (2mV AC and 8mV DC applied) showing switching between a high and a low resistance value as the cobalt magnetization is reversed by aligning with the applied magnetic field. The hysteresis is expected for a Cobalt magnetic polarizer. The magnetic field is oriented parallel to the basal (0001) plane, as indicated in the diagram (inset). Note that a single resistance jump is observed, indicating magnetization of the ribbon itself.

SI14. *Prediction of electronic conductance in graphene ribbons with side-gates*

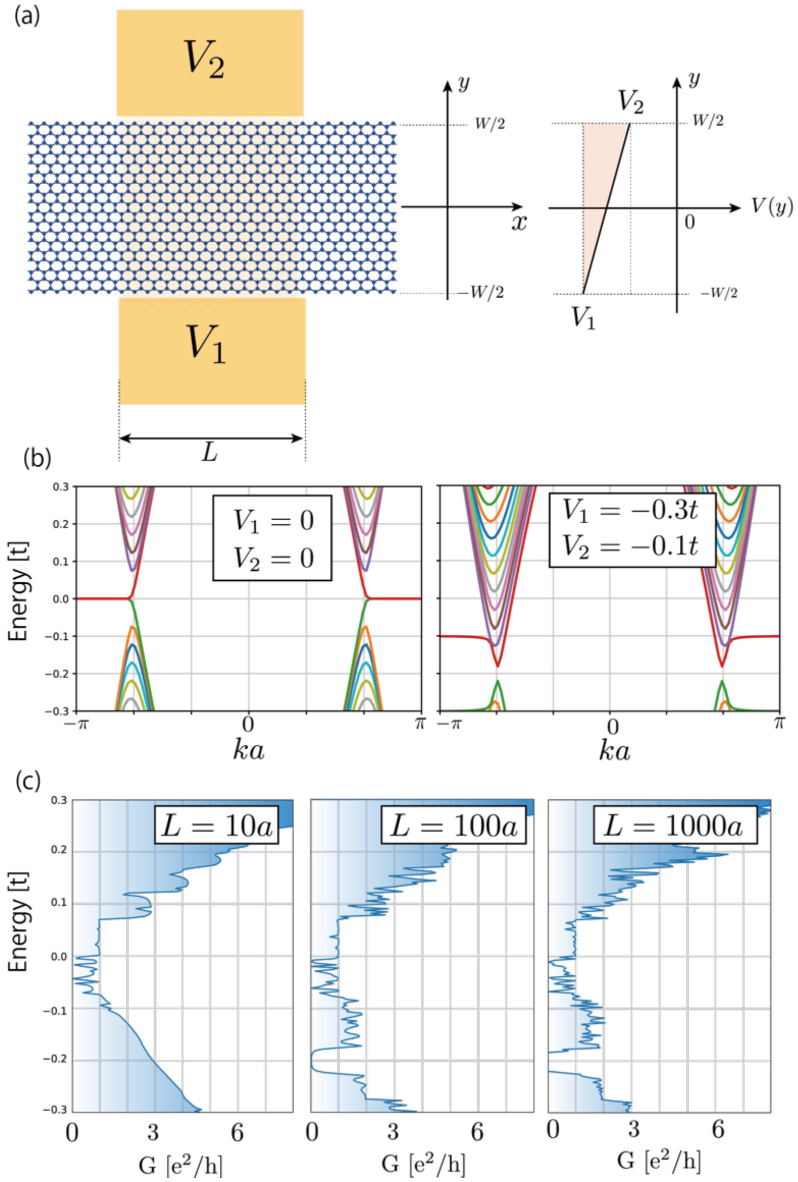

Figure S17. *Theoretical calculations for electronic conductance of graphene ribbons with side-gates*, on the basis of nearest-neighbor tight-binding model, showing that the quantum interference based top-gating effect of the edge state, found in Ref. [15] also applies to side gates. **(a)** Schematic setup of graphene ribbons with side gate. The transverse electric field is locally applied by using side gates, where lower and upper gates have bias voltages of V_1 and V_2, respectively. In this model, the linear slope potential with $V(y) = \left[\frac{(V_2-V_1)}{W}y + \frac{V_1+V_2}{2}\right]t$ is included. Here $t = 2.7$ eV is the transfer integral between nearest-neighbor carbon atoms of tight-binding model, W is the ribbon width and L is the length of side gates. **(b)** Energy band structure of ribbon with *W*=53*a*, where $a = 0.246$ nm is the lattice constant of graphene. (left) Energy band structure in absence of side gate voltage, i.e. $V_1 = V_2 = 0$. Flat bands appear owing to the edge localized states at zero energy. (right) Energy band structure in presence of side gate voltage, i.e. $V_1 = -0.3t$, $V_2 = -0.1t$. Under side gate bias, the energy bands lift downwards and opening small gap at Dirac cones. **(c)** Landauer conductance through graphene ribbons with side gate bias for several different length of side gates, i.e. (left) *L*=10*a*, (middle) 100*a* and (right) 1000*a*. The parameters for side gate bias are $V_1 = -0.3t$, $V_2 = -0.1t$. Ribbon width *W*=53*a*.

SI15. ***First observation of the edge state in transport***

Graphene research was originally motivated by the imminent need for a successor for silicon for nanoelectronics.[16,17] Earlier work on carbon nanotubes demonstrated spectacular ballistic transport properties,[18] and theory predicted that the edge state in graphene nanoribbons could have similar ballistic properties.[19-21] In fact, in graphene nanostructures the 1d edge state dominates the electronic properties and the 2d bulk does not play an essential role.

Epigraphene electronics research, pioneered at the Georgia Institute of Technology,[22-24] was motivated by these considerations and has developed independent of exfoliated graphene research. In fact, it predates exfoliated graphene by several years[22,25]. From the outset, and even now, only epigraphene (graphene grown on electronics grade silicon carbide crystals) provides the alignment and stability that is ideal for nanoelectronics fabrication.[26] In contrast lithographically patterned exfoliated graphene invariably has highly disordered graphene edges[27-29] which effectively makes it unsuited for nanoelectronics.

Since 2008 epigraphene electronics research has focused on sidewall ribbons that were grown by the thermal sublimation method on the natural and lithographically produced steps on the (0001) face of 4H-SiC and methods were developed to produce large arrays of epigraphene transistors.[30-32]

The 1 G_0 edge state was first observed in natural steps on 4H SiC in a natural sidewall that connected two lithographically graphene contacts (Fig. S15a, b).[31,32] Room temperature 1 G_0 conductance was confirmed in experiments of sidewall graphene ribbons where the conductance was measured as a function of length using an AFM probe (Fig. S15c-e).[31,32] Large arrays of graphene sidewall ribbons were produced[30,33] and the 1 G_0 edge state conductance was definitively confirmed using 4-probe methods in UHV (Fig. S16).[34]

Unexpectedly, 1 G_0 ballistic conductance is also observed meandering natural ribbons[31,32,34] (Fig. S15a) which implies that the general chiral ribbons also support the edge state. This led to the conclusion that the high temperature annealing prefers acene edges thereby significantly enhancing their applications potential.

The sidewall graphene was also found to be charge neutral in contrast to the highly charged graphene ($n \approx 10^{13}$ cm^{-2}) on the polar faces.[31,32,34] Since such large charge densities are challenging for graphene nanoelectronics in general, we initiated research on non-polar face graphene where we could use standard nanolithography methods on 2d graphene.

However, the required SiC wafers with surfaces corresponding to the sloping sidewall facets are not commercially available, so that we initiated a SiC production facility at the Tianjin International Center for Nanoparticles and Nanosystems.

Epigraphene grown on non-polar surfaces is indeed epitaxial and charge neutral as shown here (see also Fig. S14). Moreover, surprisingly, we also found that the graphene ribbons again had an edge state with a 1 G_0 conductance even without applying a high temperature annealing step. It was clear

that the plasma etching itself not only cut the graphene but also annealed the edges so that the annealing step is not required. Moreover, the 2d epigraphene itself has a low mobility which is of no consequence for the 1d edge state. These spectacular results clearly show a route to edge state 1d nanoelectronics. Moreover, as we show here, the edge state is pinned at E=0 due to the 0-DoS at E=0. This adds another unexpected aspect to epigraphene electronics and furthermore sheds light on the edge state itself which may turn out to be a Majorana fermion.

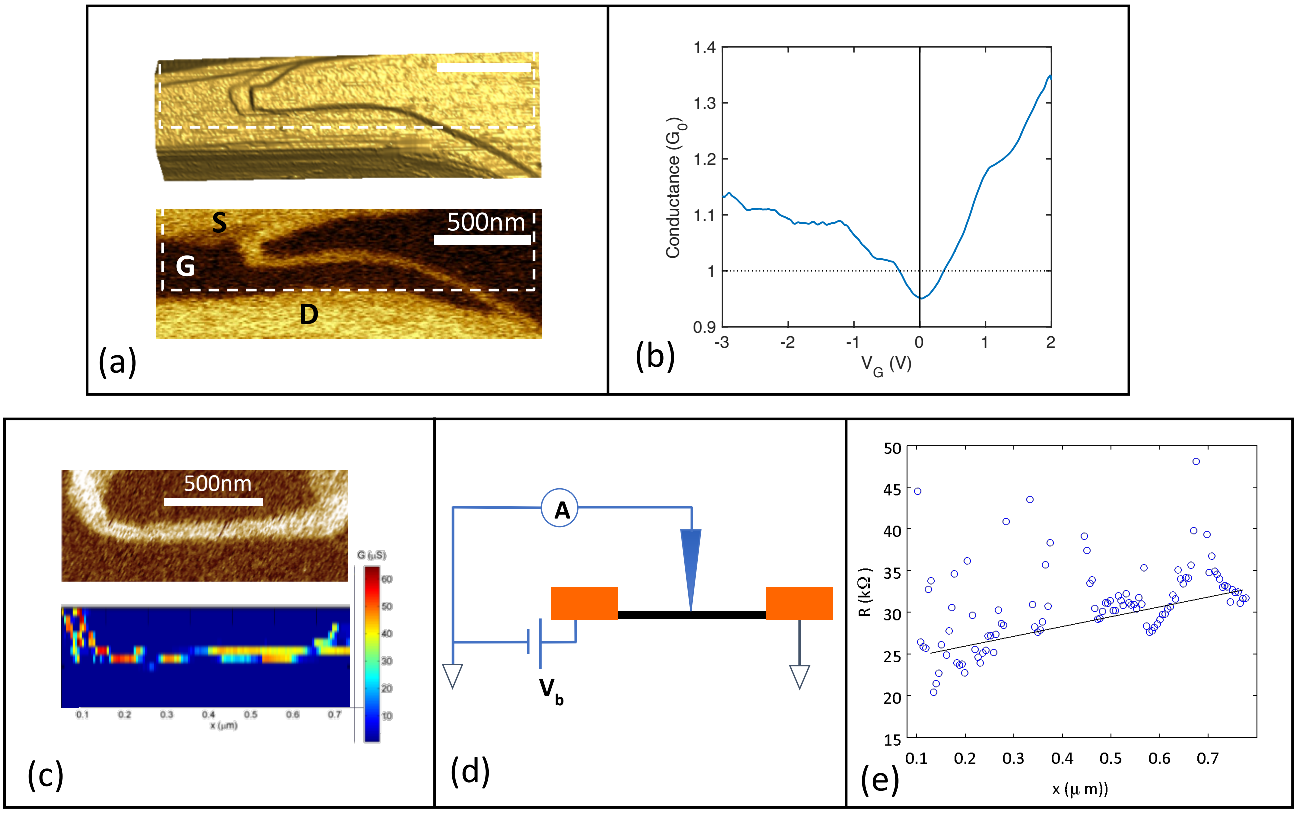

Figure S18. *First observations of the edge state*[31,32] **(a)** Top: AFM image of meandering natural step on the (0001) face of 4H-SiC. Bottom: EFM image of the same step; bright regions correspond to graphene, large bright areas are prepatterned graphene source (S) and drain (D) pads, narrow bright line is sidewall graphene decorating the step edge; white dashed line indicates outline of the gate (G). **(b)** Conductance measurements of device in (a) at T=4.5 K, showing the 1 G_0 edge state conductance **(c)** Top: EFM of natural graphene sidewall ribbon between two prepatterned graphene pads. Bottom: Local conductance trace using a conducting AFM tip as in (d). **(d)** Schematic diagram of the electrical measurement method of the device using a conducting AFM tip (blue arrow). Orange rectangles represent the graphene pads, black line represents the sidewall ribbon. **(e)** Resistance measurements, x is the distance of the tip to the left pad. The resistance is <2 kΩ when the tip is placed on the left pad. The black line is a linear fit to the data.

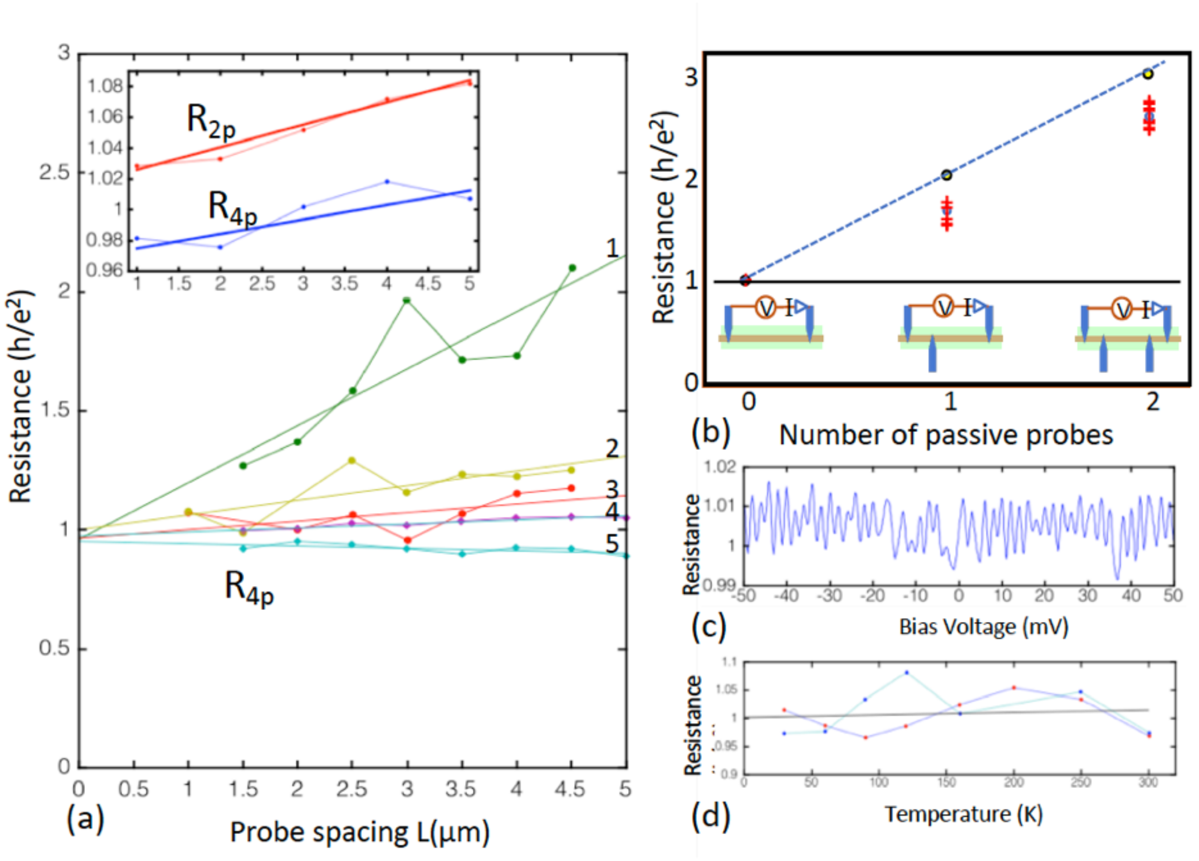

Figure S19. *Multi probe in-situ transport measurements of several 40 nm wide graphene sidewall ribbons* (adapted from Ref.[34]). **(a)** Resistances as a function of voltage probe spacing L. Linear fits extrapolate to 1 R_0 within a few percent at L=0. Slopes from 1 to 5 correspond to mean free paths λ=4.2, 28, 16, 58, >70 μm, respectively. (Inset) Sidewall ribbon with λ=106 μm; two-point measurement (red) and 4-point measurement (blue) differ by (only) 4%, indicating a probe contact resistance ≈500 Ω. **(b)** Segmentation of a sidewall ribbon caused by scattering at non-current carrying passive probes placed on the ribbon. A single non-current carrying passive probe, approximately doubles the 2-point resistance of sidewall ribbon. Two passive probes approximately triple the resistance consistent with ballistic transport theory.[12] **(c)** Resistance as a function of bias voltage V_b showing essentially no effect for -50 mV≤V_b≤50 mV **(d)** Resistance as a function of temperature for two 5 μm long ribbons showing no significant temperature dependence.

SI16. ***Effect of annealing on epigraphene nanoribbons***

Following Eq. 1 in the main text, $G(L)=1/R(L)= G_{edge} + G_{bulk}= G_e(1+L/\lambda_{edge})^{-1} + \sigma W/L$. **For a diffusive ribbon with a ballistic edge state, the resistance as a function of length of a nanoribbon provided with increasingly spaced probes will intercept the origin in absence of an edge state and will intercept at R=1 R_0 for the edge state with λ_{edge}>>L. The significant slope change after high temperature annealing indicates a decrease in the resistivity of the bulk. The concomitant increase in the L=0 intercept resistance indicates an increase in the edge state mean free path.**

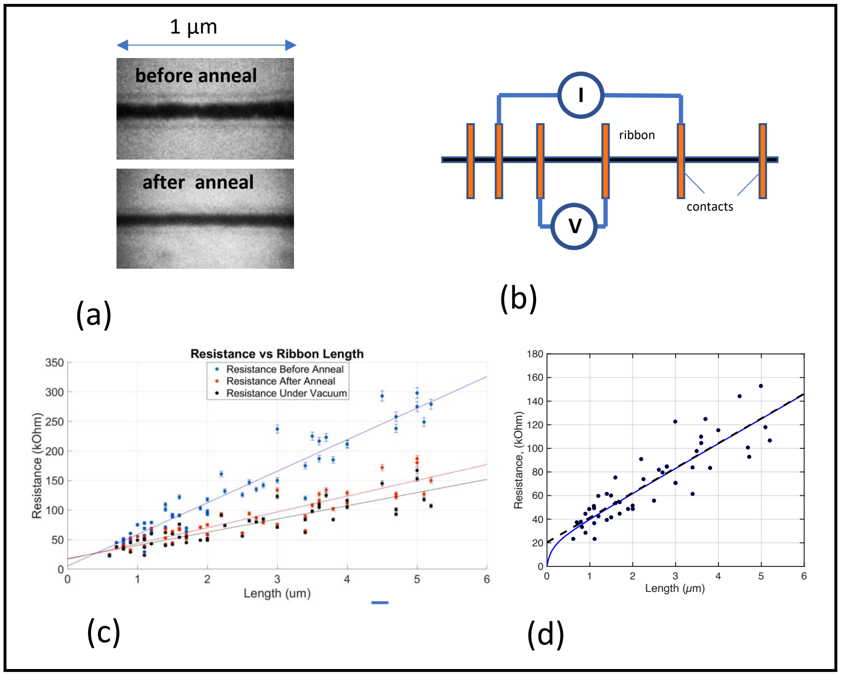

Figure S20. *Room temperature 4-point probe station resistance measurements R(L) of 90 ± 10 nm wide oxygen plasma etched Si-face graphene nanoribbons (on the Si-face) before (charge density ≈5 x 10^{12} cm^{-2}) and after vacuum annealing* at 1200°C for 15 minutes[35] **(a)** SEM image of graphene a ribbon before annealing (top) and after annealing (below), showing that annealing significantly smooths the edges and also slightly narrows the ribbon. **(b)** Schematic diagram of the device showing a nanoribbon (black) that is crossed with contact strips (orange) spaced at various intervals L, on which probes are placed for 4-point measurement. Voltage measurements are performed on single segments (uninterrupted by probes) **(c)** 4-point resistances of 50 sections at various lengths made of the unannealed device R_1, (blue); after annealing and measured in air, R_2 (orange); after annealing and measured in vacuum, R_3 (black); The linear fits are (R in units of h/e^2 and L in µm): $R_1(L) = 53.38(\pm3.21) \times L + 5.51(\pm4.23)$; $R_2(L) = 26.7(\pm2.84) \times L + 16.76(\pm2.39)$; $R_3(L) = 22.31(\pm2.29) \times L + 18.01(\pm2.56)$. More quantitatively, **(d)** shows the same annealed ribbon data as the black squares in **(b)**. The fit given by the blue line (Eq.1) is consistent with G_{edge} =1 G_0, λ_{edge}=1.1 µm, and λ_{bulk}=4 nm. The dashed line is a linear fit to the data, similarly to (a), showing that Eq.1 is dominated by the edge contribution at large length.

The data[35] in Fig. S20 clearly demonstrate the efficacy of annealing and are consistent with a 1 G_0 edge state in these narrower conventionally patterned ribbons. The relatively large spread in the

data is due to charge inhomogeneities and variations in the ribbon widths. These measurements were performed on significantly charged Si-face substrates rather than non-polar substrates at low charge densities, which contributes to relatively small λ_{edge}. Further studies on charge neutralized Si face substrates are ongoing.

SI17. *Possible low energy edge structures*

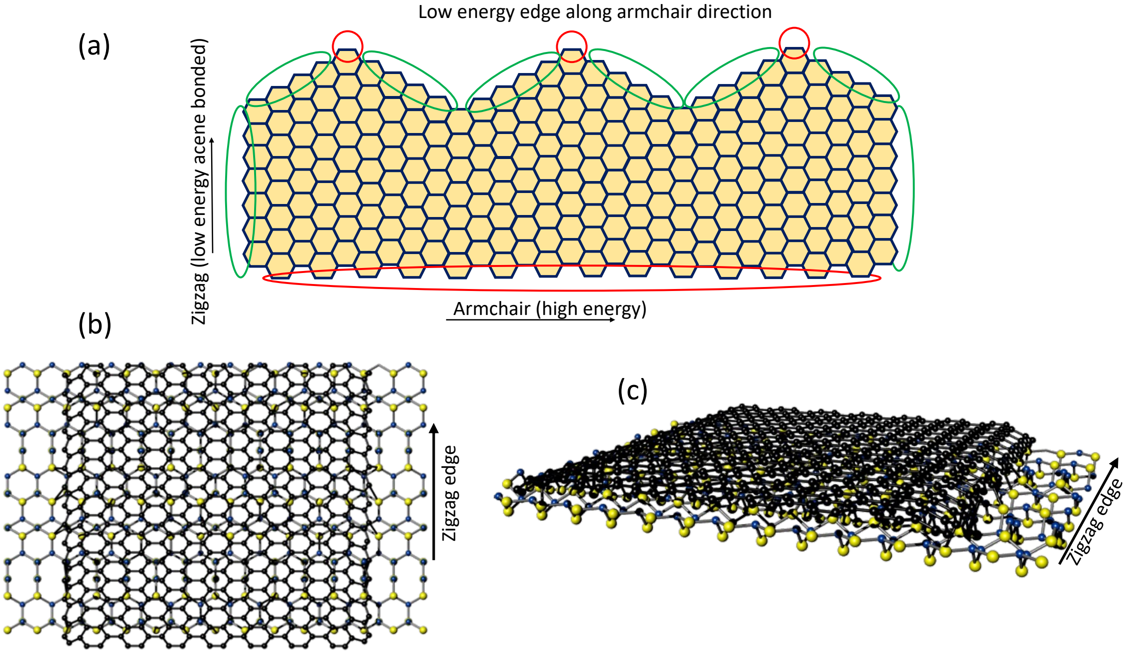

Figure S21. (a) *Schematic drawing of a nominally armchair ribbon with effective mixed edge chirality (here mostly zigzag). (b) top view and (c) perspective view of a ball and stick model of a zigzag ribbon on a bulk terminated 4H-SiC(1-105) facet (only top SiC bilayer shown). The graphene alignment corresponds to that of the Si(0001) face per continuity. The bonds between the graphene edge and SiC are hand drawn and for illustration only. A realistic structure should be fully calculated and account for surface reconstruction, which is far beyond the scope of this paper.*

Since the graphene edges are bonded to the substrate, these bond energies play an essential role in the morphology of the edge. Cross sectional TEM images of side wall ribbons show that the zigzag edges bond to the silicon and/or carbon atoms of the substrate.[36,37] The images indicate that the graphene bends down at the edge so that these bonds are perpendicular to the SiC surface and match up with the direction of the Si and C bonds of the SiC at the surface. These strong bonds passivate and mechanically stabilize the edge. In contrast, the dangling edge atoms of an armchair edge will not naturally line up with the substrate Si and C dangling bonds, so that these bonds will be strained and therefore of higher energy. If this is the case, then an annealed edge along the armchair direction will reconstruct to maximize the number of zigzag edge atoms (acene edge atoms), as schematically shown above. Hence, the nominal armchair edge can be seen as a string of zigzag edges, which is similar to a general chiral edge, which a 0-DoS peak and an edge state as shown by Beenakker et al. [38]

Supplementary References

1 Das Sarma, S., Adam, S., Hwang, E. H. & Rossi, E. Electronic Transport in Two-Dimensional Graphene. *Review of Modern Physics* **83**, 407-466 (2011).

2 Martin, J. *et al.* Observation of electron-hole puddles in graphene using a scanning single-electron transistor. *Nature Physics* **4**, 144-148 (2008).

3 Chen, J. H. *et al.* Charged-impurity scattering in graphene. *Nature Physics* **4**, 377-381 (2008).

4 Trushin, M. & Schliemann, J. Minimum Electrical and Thermal Conductivity of Graphene: A Quasiclassical Approach. *Physical Review Letters* **99**, 216602 (2007).

5 Novoselov, K. S. *et al.* Two-dimensional gas of massless Dirac fermions in graphene. *Nature* **438**, 197-200 (2005).

6 Cho, S. & Fuhrer, M. S. Charge transport and inhomogeneity near the minimum conductivity point in graphene. *Physical Review B* **77**, 081402 R (2008).

7 Tan, Y. W. *et al.* Measurement of scattering rate and minimum conductivity in graphene. *Physical Review Letters* **99**, 246803 (2007).

8 Stolyarova, E. *et al.* High-resolution scanning tunneling microscopy imaging of mesoscopic graphene sheets on an insulating surface. *Proceedings of the National Academy of Sciences* **104**, 9209 (2007).

9 Miller, D. L. *et al.* Observing the Quantization of Zero Mass Carriers in Graphene. *Science* **324**, 924-927 (2009).

10 Chen, J. H., Jang, C., Xiao, S. D., Ishigami, M. & Fuhrer, M. S. Intrinsic and Extrinsic Performance Limits of Graphene Devices on SiO2. *Nat Nanotechnol* **3**, 206-209 (2008).

11 Nguyen, T. T. N. *et al.* Topological Surface State in Epitaxial Zigzag Graphene Nanoribbons. *Nano Lett* **21**, 2876-2882 (2021).

12 Datta, S. *Electronic transport in mesoscopic systems*. (Cambridge University Press, 1995).

13 Hankinson, J. *Spin dependent current injection into epitaxial graphene nanoribbons* PhD thesis, PhD - Georgia Institute of Technology, (2015).

14 Huan, C. *et al.* Tunnel magnetoresistance of magnetic junctions based on side-wall epitaxial graphene nanoribbons. *APS March meeting*, B7.00001 (2013).

15 Wakabayashi, K. & Aoki, T. Electrical conductance of zigzag nanographite ribbons with locally applied gate voltage. *Int J Modern Physics B* **16**, 4897-4909 (2002).

16 Chen, A., Hutchby, J., Zhirnov, V. & Bourianoff, G. *Emerging Nanoelectronic Devices*. (Wiley publisher, 2014).

17 Geim, A. K. & Novoselov, K. S. The rise of graphene. *Nature Materials* **6**, 183 (2007).

18 Frank, S., Poncharal, P., Wang, Z. L. & de Heer, W. A. Carbon nanotube quantum resistors. *Science* **280**, 1744-1746 (1998).

19 Fujita, M., Wakabayashi, K., Nakada, K. & Kusakabe, K. Peculiar localized state at zigzag graphite edge. *J Phys Soc Jpn* **65**, 1920-1923 (1996).

20 Nakada, K., Fujita, M., Dresselhaus, G. & Dresselhaus, M. S. Edge state in graphene ribbons: Nanometer size effect and edge shape dependence. *Physical Review B* **54**, 17954-17961 (1996).

21 Wakabayashi, K., Takane, Y. & Sigrist, M. Perfectly conducting channel and universality crossover in disordered graphene nanoribbons. *Physical Review Letters* **99**, 036601 (2007).

22 de Heer, W. A., Berger, C. & First, P. N. Patterned thin films graphite devices and methods for making the same. *US patent US7015142B2 (Provisional filed Jun. 12, 2003). This experimentally supported patent provides the foundation of graphene nanoelectronics in general and expliclty describes seamless zero-mode epigraphene electronics.*

23 Berger, C. *et al.* Ultrathin Epitaxial Graphite: 2D Electron Gas Properties And a Route Toward Graphene-Based Nanoelectronics. *J Phys Chem B* **108**, 19912-19916 (2004).

24 Berger, C. *et al.* Electronic confinement and coherence in patterned epitaxial graphene. *Science* **312**, 1191-1196 (2006).

25 de Heer, W. A. Patterned Graphite Nanoelectronics. *NSF proposal 01-157* **12/19/2001**, https://graphene.gatech.edu/historicalpersp.html (2001).

26 Berger, C., Conrad, E. & de Heer, W. A. in *Physics of Solid Surfaces, Landolt Börstein encyclopedia* Vol. Subvolume B *Physics of Solid Surfaces* (ed P. Chiaradia G. Chiarotti) Ch. 164-171, 727-807. ArXiv:1704.00374 (Springer-Verlag, 2018).

27 Epping, A. *et al.* Insulating State in Low-Disorder Graphene Nanoribbons. *physica status solidi (b)* **256**, 1900269 (2019).

28 Stampfer, C. *et al.* Energy Gaps in Etched Graphene Nanoribbons. *Physical Review Letters* **102**, 056403 (2009).

29 Gallagher, P., Todd, K. & Goldhaber-Gordon, D. Disorder-induced gap behavior in graphene nanoribbons. *Physical Review B* **81**, 115409 (2010).

30 Sprinkle, M. *et al.* Scalable templated growth of graphene nanoribbons on SiC. *Nat Nanotechnol* **5**, 727-731 (2010).

31 Ruan, M. *Structured epitaxial graphene for electronics* PhD thesis, PhD - Georgia Institute of Technology, (2012).

32 Ruan, M. *et al.* Epitaxial graphene on silicon carbide: Introduction to structured graphene. *MRS Bulletin* **37**, 1138-1147 (2012).

33 Hicks, J. *et al.* A wide-bandgap metal-semiconductor-metal nanostructure made entirely from graphene. *Nature Physics* **9**, 49-54 (2013).

34 Baringhaus, J. *et al.* Exceptional ballistic transport in epitaxial graphene nanoribbons. *Nature* **506**, 349-354 (2014).

35 Hu, Y. *The edge states of epitaxial graphene on SiC, PhD dissertation* PhD dissertation thesis, Georgia Tech, (2021).

36 Palacio, I. *et al.* Atomic Structure of Epitaxial Graphene Sidewall Nanoribbons: Flat Graphene, Miniribbons, and the Confinement Gap. *Nano Lett* **15**, 182−189 (2015).

37 Norimatsu, W. & Kusunoki, M. Growth of graphene from SiC{0001} surfaces and its mechanisms. *Semicond Sci Tech* **29**, 064009 (2014).

38 Akhmerov, A. R. & Beenakker, C. W. J. Boundary conditions for Dirac fermions on a terminated honeycomb lattice. *Physical Review B* **77**, 085423 (2008).
